# Thromboxane A_2_ exacerbates acute lung injury via promoting edema formation

**DOI:** 10.1038/srep32109

**Published:** 2016-08-26

**Authors:** Koji Kobayashi, Daiki Horikami, Keisuke Omori, Tatsuro Nakamura, Arisa Yamazaki, Shingo Maeda, Takahisa Murata

**Affiliations:** 1 Department of Animal Radiology, Graduate school of Agriculture and Life Sciences, The University of Tokyo, Japan

## Abstract

Thromboxane A_2_ (TXA_2_) is produced in the lungs of patients suffering from acute lung injury (ALI). We assessed its contribution in disease progression using three different ALI mouse models. The administration of hydrochloric acid (HCl) or oleic acid (OA)+ lipopolysaccharide (LPS) caused tissue edema and neutrophil infiltration with TXA_2_ production in the lungs of the experimental mice. The administration of LPS induced only neutrophil accumulation without TXA_2_ production. Pretreatment with T prostanoid receptor (TP) antagonist attenuated the tissue edema but not neutrophil infiltration in these models. Intravital imaging and immunostaining demonstrated that administration of TP agonist caused vascular hyper-permeability by disrupting the endothelial barrier formation in the mouse ear. *In vitro* experiments showed that TP-stimulation disrupted the endothelial adherens junction, and it was inhibited by Ca^2+^ channel blockade or Rho kinase inhibition. Thus endogenous TXA_2_ exacerbates ALI, and its blockade attenuates it by modulating the extent of lung edema. This can be explained by the endothelial hyper-permeability caused by the activation of TXA_2_-TP axis, via Ca^2+^- and Rho kinase-dependent signaling.

Acute lung injury (ALI) and its severe manifestation, acute respiratory distress syndrome (ARDS), are lethal and complex respiratory dysfunctions that includes various pathogenic factors such as aspiration of gastric contents, microbial infection, sepsis, and trauma^1^,^2^. There are two major pathological features of ALI/ARDS; edema and neutrophil accumulation in the lung tissue. Initial inflammatory stimuli disrupt lung endothelial and/or epithelial barrier and induce extravasation of protein rich fluid resulting in lung edema. These stimuli also cause neutrophil infiltration into the interstitium and alveolar airspace. Infiltrated neutrophils injure lung parenchymal cells by secreting elastase and reactive oxygen species, inducing the further production of pro-inflammatory cytokines, and activation of inflammatory cells^3^. These physical and chemical tissue damages lead to the impairment of air exchange and severe respiratory dysfunction. Although many studies have focused on the mechanisms underlying endothelial/epithelial barrier disruption and neutrophil accumulation upon inflammation, there is a lack of an integrated understanding of these complex diseases. Although clinical research shows that the treatment with an anti-inflammatory steroid methylprednisolone reduces mortality in ARDS patients^4^, there are limited clinical treatments available. Thus, it is urgently needed that a better understanding of ALI/ARDS pathology and development of a novel therapeutic strategy, which take into consideration each pathogenesis and progression stage.

There are several experimental mouse models that are currently used to mimic human ALI/ARDS. Intratracheal instillation of hydrochloric acid (HCl) directly injures epithelial cell and endothelial cell in mouse lungs. These damages lead both tissue edema and neutrophil accumulation in the lungs^5^. This model mimics human ALI/ARDS induced by aspiration of gastric juice contents. Intravenous injection of oleic acid (OA) injures endothelial cells by inhibiting the Na^+^-K^+^-ATPase, causing severe lung edema but not apparent neutrophil accumulation in mice^6^. This model is used to mainly mimic lipid embolism-induced human ALI/ARDS. Intravenous or intratracheal instillation of lipopolysaccharide (LPS) stimulates cytokine secretion from alveolar macrophages, and expression of endothelial adherens molecules in mouse lungs. This model reproduces sepsis-associated ALI/ARDS characterized by severe neutrophil accumulation in the lungs^7^. Thus, to investigate the mechanism of disease progression, it is necessary to select the appropriate model, which takes into consideration the disease manifestation as well as the pathways involved in ALI.

Inflammatory lipid mediators, prostanoids (PGs), are produced enzymatically by the activation of cyclooxygenase (COX) and prostaglandin/thromboxane synthases from fatty acids. There are five major PGs; prostaglandin D_2_ (PGD_2_), PGE_2_, PGF_2α_, PGI_2_ and thromboxane A_2_ (TXA_2_) which are strongly involved in the inflammatory response. Previous studies have focused on the contribution of COX and PGs in ALI/ARDS^8^,^9^,^10^,^11^. Elevated levels of PGs have been reported in bronchoalveolar lavage (BAL) fluid obtained from patients with ARDS^12^. Hinshaw *et al.* originally found that COX inhibition prevented the development of sepsis and improved survival rates in dogs^8^. Another group reported that in mouse transfusion-related ALI/ARDS model, treatment with a COX inhibitor, aspirin, ameliorated lung edema and increased the survival rate^9^. These findings suggest that COX-mediated production of PGs is crucial for the initiation and progression of lung inflammation. However, in a clinical study, treatment with a nonselective COX inhibitor ibuprofen did not reduce the incidence of ARDS in patients with sepsis^10^. These observations suggest that PGs play a multifaceted role, being both pro-inflammatory and/or anti-inflammatory mediators in the pathophysiology of airway inflammation. To reveal this complexity, detailed evaluation of the role of each PG using multiple models is indispensable.

Previous studies revealed that major PGs including PGE_2_ and PGI_2_ act mostly as anti-inflammatory PGs in ALI/ARDS. Treatment with the PGE_2_ or PGI_2_ analog, beraprost, reduces the increase in BAL cell count and the extravasation of BAL proteins in the ventilator-related ALI mouse model^13^. Anti-inflammatory roles of lipoxin A_4_ or 15-deoxy Δ12,14 prostaglandin J_2_ have been reported in mouse HCl- or carrageenin-induced ALI/ARDS models, respectively^14^,^15^. We also reported that deficiency of hematopoietic PGD synthase accelerates, and stimulation of PGD_2_ receptor inhibits induction of edema and neutrophil accumulation in the lungs of LPS-induced ALI/ARDS model mice^16^. Given that the pathophysiological action of each PG varies with its target cells, context of activation, and pathogenesis, a detailed evaluation of the role of each PG using multiple models is indispensable to reveal this complexity and to overcome ALI/ARDS.

TXA_2_ is one of the prostanoids generated in several cell types such as platelets, monocytes, macrophages, and epithelial cells^17^. TXA_2_ is produced by the action of COX, followed by thromboxane synthase (TXS). TXA_2_ binds to a GPCR, T prostanoid (TP) receptor, that couples to the G_q_ or G_12/13_ signaling molecules^18^. TP stimulation is known to cause a broad range of cellular responses such as platelet aggregation and vasoconstriction^18^. In the inflamed lungs of ALI/ARDS patients, TXA_2_ as well as the other PGs mentioned before, were detected^19^. Experimental studies have suggested that TXA_2_ has a pro-inflammatory role in lung inflammation^20^,^21^,^22^. The treatment with a TXA_2_ synthase inhibitor, ozagrel, restored the impaired respiratory function and decreased arterial O_2_ pressure in guinea-pig ALI induced by OA^21^. The treatment with a TP antagonist, SQ29548, improved the symptoms of HCl-induced mouse ALI/ARDS^22^. Thus, TXA_2_ has been suggested to be involved in the progression of ALI/ARDS. However, it remains unclear how TXA_2_-signaling exacerbates ALI/ARDS.

We investigated the role of TXA_2_-signaling in three different models of ALI/ARDS. We found that production of TXA_2_ varies according to the pathogenesis of ALI and its production positively correlates with induction of edema, but not with neutrophil accumulation in inflamed lungs. Mechanistically, TXA_2_ stimulates the endothelial TP receptor and disrupts endothelial barrier via Ca^2+^/Rho kinase-signaling. These findings reveal pathophysiological implications of TXA_2_ and might provide novel therapeutic targets for treating ALI.

## Results

### TXA_2_ exacerbates HCl-induced ALI

**Figure 1:**
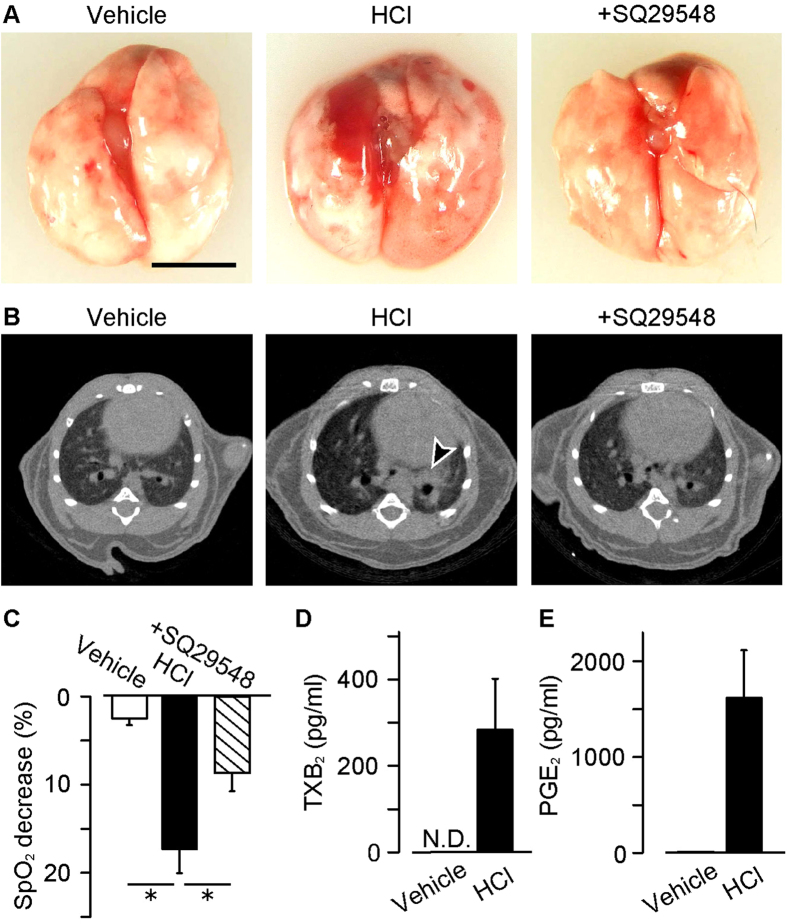
TXA_2_ abrogates HCl-induced lung inflammation via stimulating TP receptor. SQ29548 (i.p., 2 mg/kg, 0, 2 and 4 h after HCl administration) was administered to the HCl (intra-nasally, 0.1 M, 2.5 μl/g, 6 h)-treated mice. (**A**) Representative pictures of the inflamed lungs (n = 8–14). Bar, 5.0 mm. (**B**) Representative pictures of CT scan (n = 5–8). (**C**) Values of saturation of peripheral oxygen (SpO_2_) in the mice (n = 6–12). (**D**) TXA_2_ content in lung BAL fluid (n = 5). (**E**) PGE_2_ content in lung BAL fluid (n = 5). Data are presented as mean ± SEM. *P < 0.05.

We first investigated the role of TXA_2_ in the acid aspiration-induced ALI model. The administration of HCl (intra-nasally, 0.1 M, 2.5 μl/g, 6 h) caused severe hemorrhage in the lungs of the mice (Fig. 1A). In these mice, infiltrative shadow indicating tissue inflammation was observed, especially around bronchi in computed tomography (CT) imaging (Fig. 1B, indicated by arrowhead). Treatment with a TP receptor antagonist (SQ29548, i.p., 2 mg/kg, 0, 2 and 4 h after HCl administration) significantly inhibited these features. The HCl administration also impaired respiratory function indexed as the value of saturation of peripheral oxygen (SpO_2_, Fig. 1C). The treatment with SQ29548 significantly recovered the HCl-induced respiratory dysfunction.

We next measured the amount of a stable TXA_2_ metabolite TXB_2_ in BAL fluids. In the vehicle-administered mice, no TXB_2_ was detected, while 284 ± 118 pg/ml of TXB_2_ was detected in the HCl-administered mice (Fig. 1D). The HCl-administration also increased the production of PGE_2_ (Fig. 1E) but it did not change the amount of a PGI_2_ metabolite, 6-keto PGF1α in the BAL fluid (Fig. S1).

These results suggested that the HCl-administration stimulates TXA_2_-TP signaling which leads to severe inflammation and impaired respiratory function.

### TXA_2_-TP signaling activation causes lung edema in HCl-induced ALI

**Figure 2:**
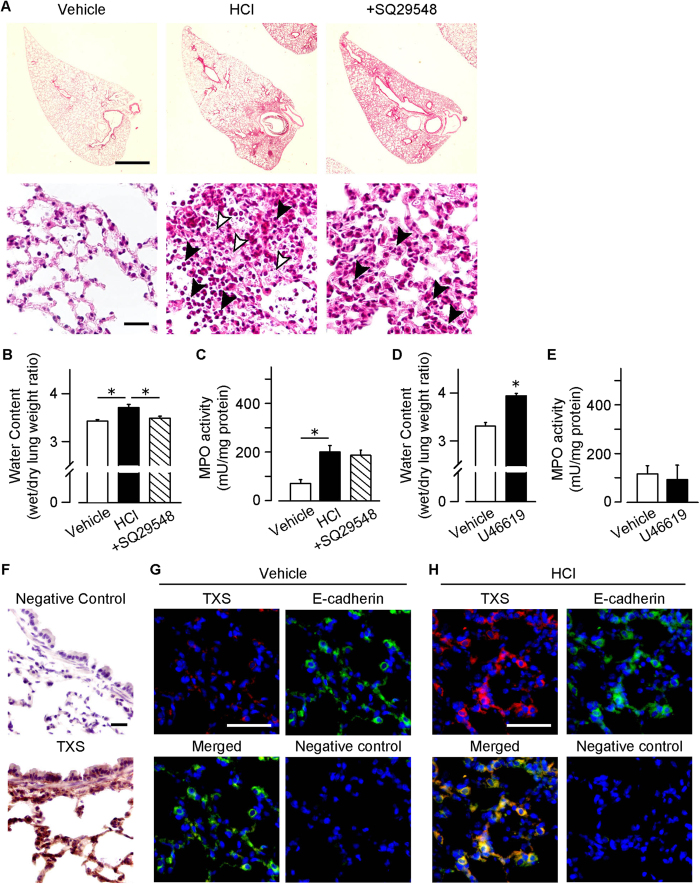
TXA_2_-TP signaling abrogates the lung edema formation. SQ29548 (i.p., 2 mg/kg, 0, 2 and 4 h after HCl administration) was administered to the HCl (intra-nasally, 0.1 M, 2.5 μl/g, 6 h)-treated mice. (**A**) Representative pictures of H&E staining (n = 7). Bar, 1.0 mm or 20 μm. (**B**) Water contents of the inflamed lungs (n = 5–7). (**C**) Activities of myeloperoxidase (MPO) in the lung homogenates (n = 8). Mice were intraperitoneally administrated with U46619 (25 μg/kg, 1 h). (**D**) Water contents of the inflamed lungs (n = 6–7). (**E**) Activities of MPO in the lung homogenates (n = 4–5). (**F**) Representative pictures of immunostaining of TXS in the HCl-treated lungs (lower panel, n = 4). Upper panel shows negative controls which only treated with secondary antibody Bar, 20 μm. (**G,H**) Representative pictures of immunostaining of TXS (upper left panel, red) and E-cadherin (upper right panel, green) in vehicle (**G**) and HCl-treated mice (**H**, n = 4 each). Lower left panel shows merged pictures of TXS and E-cadherin staining. Lower right panel shows negative controls which only treated with secondary antibody. Bar, 50 μm. Data are presented as mean ± SEM. *P < 0.05.

Morphological studies showed that the HCl administration caused pulmonary hemorrhage and neutrophil accumulation, especially around the bronchi (Fig. 2A, neutrophils are indicated by black arrow heads in lower panels). These phenomena were accompanied with the leakage of protein rich fluid into tissue interstitium (indicated by white arrowheads). The treatment with SQ29548 reduced hemorrhage and leakage of protein rich fluid. Interestingly, neutrophil accumulation was observed even in the SQ29548-treated group.

We evaluated the effects of TXA_2_-TP signaling on two major pathological features of ALI; lung edema and neutrophil accumulation. The administration of HCl significantly increased water content, which is an index of tissue edema, and the activity of myeloperoxidase (MPO) which is an index of neutrophil infiltration (Fig. 2B). The treatment with SQ29548 almost completely suppressed edema formation. In contrast, this regimen slightly, but not significantly, reduced the HCl-induced neutrophil infiltration into the lung tissue (Fig. 2C).

We further examined whether TP agonism itself affects lung edema and neutrophil accumulation. Intraperitoneal treatment with a TP agonist (U46619, 25 μg/kg, 1 h) induced lung edema (Fig. 2D) but did not affect MPO activity in lung (Fig. 2E). These results showed that TXA_2_-TP signaling exacerbates the HCl-induced ALI via induction of edema.

### Epithelial cells produces TXA_2_ in HCl-induced ALI

We examined what type of cells produced TXA_2_ in the HCl-induced ALI. Immunohistochemistry showed that the HCl-administration induced TXS expression along alveolar walls (Fig. 2F). Consistently, immunofluorescence study showed that E-cadherin positive epithelial cells strongly express TXS in the HCl-treated mouse lung (Fig. 2G,H), suggesting that epithelial cells are the major source of TXA_2_ in the HCl-induced ALI.

### The role of TXA_2_-TP signaling in oleic acid-induced ALI

**Figure 3:**
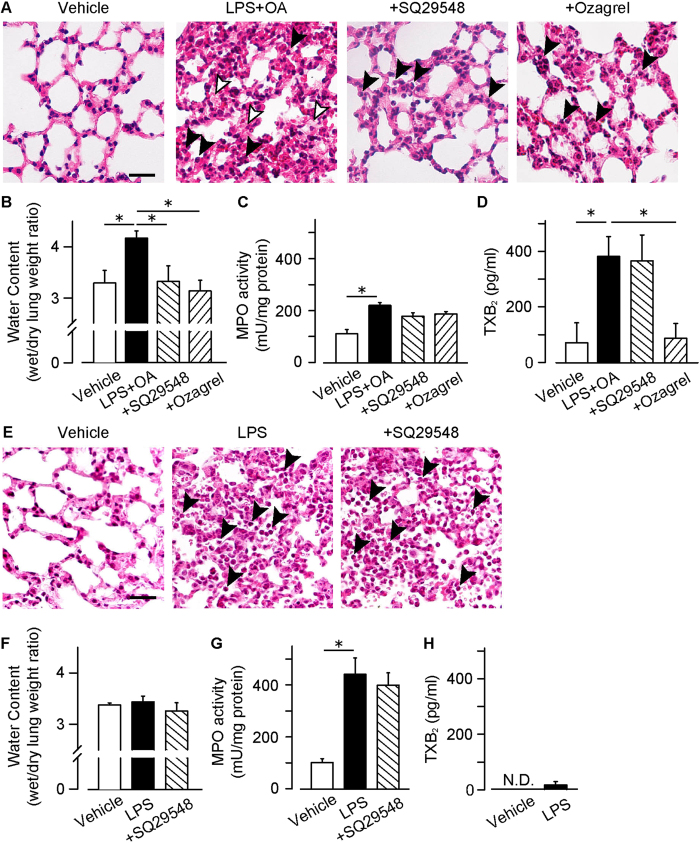
Contribution of TXA_2_-TP signaling in OA and/or LPS-induced ALIs. SQ29548 (i.p., 2 mg/kg, 0, 2 and 4 h after LPS administration) or Ozagrel (i.p., 50 mg/kg 15 min before OA administration) was administered to the OA (i.v., 150 μl/kg) and LPS (intra-nasally, 1.5 mg/kg, 6 h)-treated mice. (**A**) Representative pictures of H&E staining. Bar, 50 μm. (**B**) Water contents of the inflamed lungs (n = 7–15). (**C**) Activities of myeloperoxidase (MPO) in the lung homogenates (n = 6–15). (**D**) TXB_2_ contents in BAL fluids (n = 5). SQ29548 (i.p., 2 mg/kg, 0, 2 and 4 h after LPS administration) was administered to the LPS (intra-nasally, 3.75 mg/kg, 6 h)-treated mice. (**E**) Representative pictures of H&E staining. Bar, 50 μm. (**F**) Water contents of the inflamed lungs (n = 4). (**G**) Activities of MPO in the lung homogenates (n = 8–9). (**H**) TXB_2_ contents in BAL fluids (n = 5). Data are presented as mean ± SEM. *P < 0.05.

Oleic acid (OA) causes severe histopathological changes, similar to those observed in ARDS. Administration of OA (150 μl/kg) in addition to LPS (1.5 mg/kg) caused severe alveolar damage. At 6 h after the LPS administration, damage to the lung was characterized by extensive pulmonary hemorrhage (Fig. S2A) and broad infiltrative shadow in CT imaging (Fig. S2B). However, this stimulation did not cause respiratory dysfunction (Fig. S2C). As shown in Fig. 3A, morphological studies showed that the LPS + OA-administration caused severe hemorrhage, protein leakage (middle panel, indicated by while arrowheads), and neutrophil accumulation (indicated by black arrow heads). Consistently, tissue water content and MPO activity were significantly increased in these lungs (Fig. 3B,C). The extent of these changes was larger than that in the HCl-induced ALI model (Fig. 2B,C). As shown in Fig. 3D and Fig. S2D, LPS + OA stimulation increased the TXB_2_ content in the BAL fluid (359 ± 94 pg/ml) as well as PGE_2_. LPS + OA-induced TXA_2_ production was abolished by the pretreatment with a TXS inhibitor ozagrel (i.p., 50 mg/kg, 15 min before OA administration). Pretreatment with either SQ29548 (i.p., 2 mg/kg, 0, 2 and 4 h after LPS administration) or ozagrel effectively diminished the injuries in this model (Fig. 3A, right panels and Fig. 3B). As observed in the HCl-induced ALI model, these regimens did not rescue the neutrophil accumulation in the LPS + OA-treated lungs (Fig. 3C).

### The role of TXA_2_-TP signaling in LPS-induced ALI

We next utilized the LPS-induced lung inflammation model, which reproduces the sepsis-associated ALI/ARDS. The administration of LPS (intra-nasally, 3.75 mg/kg, 6 h) caused lung inflammation accompanied with intensive neutrophil accumulation (Fig. 3E, middle panel, indicated by black arrowheads), as well as an increase in MPO activity in the lungs (Fig. 3G). This stimulation did not cause severe hemorrhage (Fig. S3A), lung dysfunction (Fig. S3B), and pulmonary edema (Fig. 3F), which were observed in the HCl- or LPS + OA-induced ALI. The treatment with SQ29548 (i.p., 2 mg/kg, 0, 2 and 4 h after LPS administration) did not have any beneficial action against the neutrophil accumulation induced by LPS (Fig. 3E, right panel). Although the LPS-administration increased the amounts of TXB_2_ and PGE_2_ in the BAL fluid (Figs 3H and S2C), its amount (36 ± 24 and 301 ± 34 pg/ml, respectively) was much smaller than that in the HCl- or LPS + OA-treated lungs (Figs 1D,E, 3D and S2D). Thus, the production and contribution of TXA_2_ seems to be relatively lower in this model.

### TXA_2_ increases vascular permeability *in vivo*

**Figure 4:**
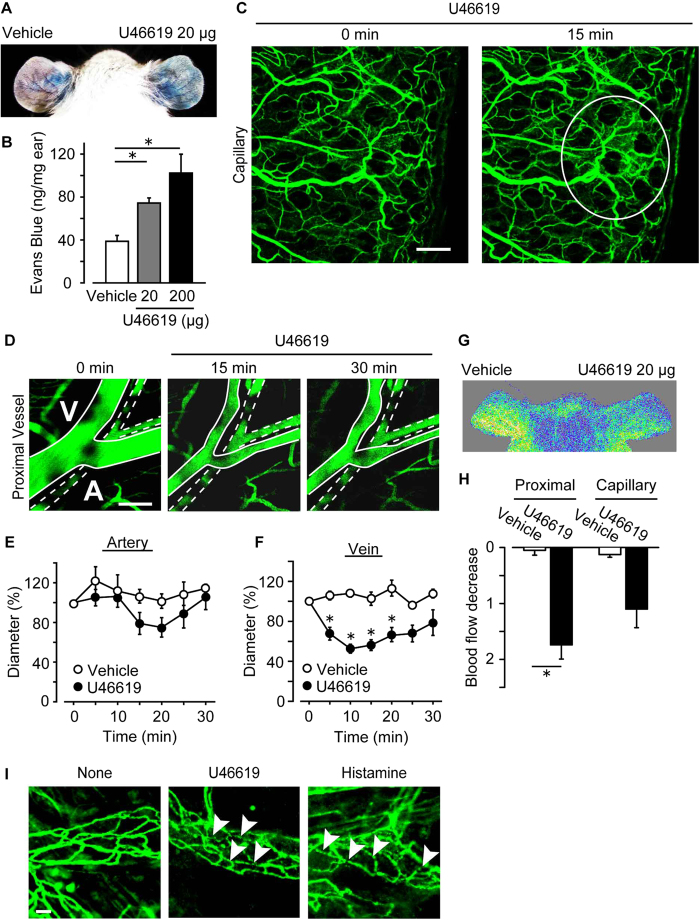
TP-stimulation causes hyper-permeability via endothelial barrier disruption *in vivo*. (**A**) Representative pictures of Miles assay. (**B**) Summary of dye extravasation (n = 4). (**C**) Representative pictures of FITC-dextran extravasation in mice capillary (n = 6). Bar, 200 μm. (**D**) Representative pictures of the vasculature in mouse ear. (**E,F**) Summary of the change of vascular diameter (n = 4–6). (**G**) Representative pictures of blood flow measurement by laser Doppler. (**H**) Summary of blood flow changes (n = 4). (**I**) Representative pictures of whole-mount immunostainings of VE-cadherin. Bar, 10 μm. Data are presented as mean ± SEM. *P < 0.05.

Sustained vascular hyper-permeability can cause tissue edema. Therefore, we investigated the effect of TXA_2_-TP signaling on vascular permeability *in vivo*. A TP agonist U46619 was applied to the right ear of mice followed by the injection of Evans Blue for 30 min. As shown in Fig. 4A (representative pictures) and 4B (summary), treatment with 20–200 μg/ear U46619 extravasated blue dye in mouse ear in a dose-dependent manner.

Ear vasculature is mainly composed of proximal vessels, distal vessels, and capillaries (Fig. S4A). In proximal vessels or distal vessels, endothelial cells are covered by vascular mural cells such as smooth muscle cells or pericytes. Capillaries are composed only of endothelial cells. Contraction of mural cells reduces blood flow which decreases the intravascular hydrostatic pressure and vascular permeability. Given that TXA_2_ is known as a vasoconstrictor, we examined the effect of TXA_2_ on vascular leakage and contraction *in vivo*. Intravital microscopy showed that the application of U46619 (20 μg/ear, 15 min) caused the FITC-dextran leakage in capillaries (Fig. 4C). However, this regimen induced contraction in veins but not significantly in arteries (Fig. 4D: representative picture, 4E and 4F: summaries). Laser Doppler blood-flow imaging showed that the application of U46619 (20 μg/ear, 20 min) significantly decreased the blood flow in mouse pinna (representative picture: Fig. 4G, summary: 4H). Thus, TP-stimulation may limit blood flow by inducing vascular contraction in mouse ear.

We next assessed the effect of U46619 on endothelial barrier by observing intercellular adherens junctions in the ear. Whole-mount immunostaining showed that an intracellular adhesion molecule, VE-cadherin, was located at cell-cell contact areas under non-stimulated conditions in venulae (Fig. 4I, left panel). Changes in VE-cadherin localization, internalization or disassembly, induces endothelial barrier disruption. The administration of U46619 (20 μg/ear, 15 min) caused partial disassembly of VE-cadherin, as indicated by the arrowheads in Fig. 4I (middle panel). Exposure to histamine (400 μg/ear, 15 min), a well-characterized endothelial barrier disruptor, also triggered VE-cadherin disassembly (Fig. 4I, right panel).

Figure S4B shows that similar results were obtained by *en face* immunostaining of mouse pulmonary artery. VE-cadherin was located at cell-cell contact areas in the absence of stimulation (left panel). The treatment with U46619 (1 μM) disassembled VE-cadherin (middle panel. indicated by black arrows heads). The treatment with histamine (10 μM) also caused VE-cadherin disassembly (right panel).

### TXA_2_ induces endothelial barrier disruption by both intracellular Ca^2+^ and Rho kinase

**Figure 5:**
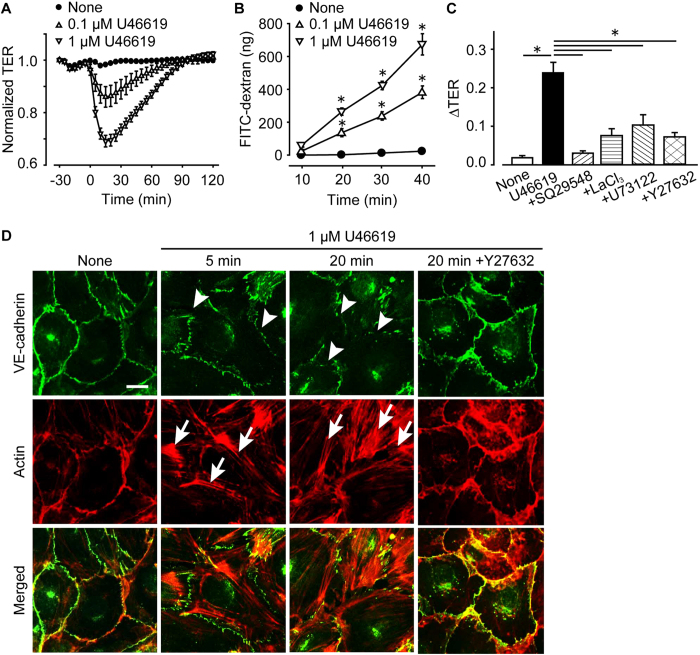
TP-stimulation induces endothelial barrier disruption by the increase in intracellular Ca^2+^ concentration and activation of Rho kinase. (**A**) TER of HUVECs (n = 10). (**B**) FITC-dextran leakage of HUVECs (n = 6). (**C**) Summary of the maximum decrease of TER. Ca^2+^ channel inhibitor (LaCl_3_, 5 μM), PLC inhibitor (U73122, 3 μM), or Rho kinase inhibitor (Y27632, 10 μM) were applied (n = 8–14). (**D**) Representative pictures of immunostaining of VE-cadherin (upper panels, green) and F-actin (middle panels, red, n = 4–6). Bottom panels show merged pictures of VE-cadherin and F-actin staining. Bar, 10 μm. Data are presented as mean ± SEM. *P < 0.05.

Endothelial barrier function was evaluated *in vitro* by measuring TER and the amount of FITC-dextran passage. Treatment with U46619 (0.1–1 μM) decreased the TER of human umbilical vein endothelial cells (HUVECs) in a dose-dependent manner, and this reduction lasted for about 50 or 60 min (Fig. 5A). Consistently, U46619 (0.1–1 μM) significantly increased the amount of dextran passage across the HUVECs monolayer (Fig. 5B). We also confirmed that U46619 (0.01–1 μM) dose-dependently decreased TER in human pulmonary artery endothelial cells (HPAECs) and human microvascular endothelial cells (HMVECs) indicating that various types of endothelial cells express TP receptor (Fig. S5). The TP blockade by SQ29548 (1 μM, 30 min pretreatment) almost completely inhibited the U46619 (1 μM)-induced decrease of TER (Fig. 5C, summary). Blockade of Ca^2+^ channel (LaCl_3_, 5 μM, 30 min pretreatment), inhibition of phospholipase C (U73122, 3 μM, 30 min pretreatment), and inhibition of ROCK (Y27632, 10 μM, 30 min pretreatment) also attenuated the reduction in TER. These *in vitro* results suggested that TP-stimulation disrupts endothelial barrier function, mainly via Ca^2+^/ROCK signaling.

Using immunostaining, we evaluated the effect of TXA_2_ on the endothelial barrier *in vitro*. In the basal condition, VE-cadherin and F-actin were aligned along cell-cell adhesion area of HUVECs indicating adherens junction formation (Fig. 5D, left panels). The administration of U46619 (1 μM, 20 min) destroyed VE-cadherin alignment (indicated by arrowheads) and induced actin stress fiber formation (indicated by arrows), which are characteristic phenomena of adherens junction disassembly. Pretreatment with Y27632 (10 μM, 30 min pretreatment) rescued the U46619-induced disruption of adherence junction (right panels).

## Discussion

Although several pharmacological reagents including corticosteroids and adrenaline β_2_ agonists are known to have a beneficial therapeutic effect on ALI and ARDS, they do not improve the mortality in large scale clinical trials^4^,^23^. As of now, only supportive treatment is available and the mortality rate of ALI is still high^24^. ALI and ARDS are complex syndromes with severe and rapidly progressing respiratory dysfunction caused by various pathogenic factors. Therefore, there is an urgent need to understand the mechanism of disease progression and develop effective and appropriate therapeutic strategies that considers the pathogenic mechanism.

Using three different ALI models, we showed that the production of a lipid mediator TXA_2_ resulted in different pathogenicity, and that TXA_2_ also contributed to lung edema by disrupting the endothelial barrier. Comparing these three models, the amounts of TXA_2_ (indexed as TXB_2_) positively correlated with the edema formation (indexed as water content, Fig. S6, left panel) but not neutrophil infiltration (indexed as MPO activity, Fig. S6, right panel). These observations highlight our conclusion that TXA_2_ is the critical factor of edema formation in ALI progression.

HCl aspiration directly injures alveolar epithelial cells and causes lung edema neutrophil accumulation and respiratory dysfunction^25^. We propose in this study, that epithelial cells express TXS and are the major source of TXA_2_ in the HCl-induced ALI. Alveolar damage by chemical and/or physical stimulation may be attributed to the TXS induction and TXA_2_ production, and subsequent edema formation in ALIs. Previous studies showed that the administration of HCl activated platelets, which in turn produced TXA_2_ resulting in lung edema^22^. However, we could neither detect significant increase in the number of platelets nor in TXS expression in platelets in our current model (Fig. S7). Further investigations are required to clarify this point.

The administration of HCl induced lung edema and neutrophil accumulation accompanied with TXA_2_ production. This stimulation impaired respiratory function. The LPS + OA challenge induced more severe manifestation of the disease with TXA_2_ production than the HCl did, while it did not cause respiratory dysfunction. This discrepancy seen between lung dysfunction and other features may be due to the difference in the pathogenic mechanisms of these two models. Aspirated HCl, but not OA directly injures and then induces necrosis of the epithelial cells which play a crucial role in gas exchange^26^. Although tissue edema, i.e. accumulation of fluid in the pulmonary interstitium, also influences gas exchange, its effects on the pathophysiology of ALI seem to be relatively small at least in the present models. This difference in the pathogenic mechanism probably reflects the responsiveness to TP antagonism. The effect of TP antagonism on lung dysfunction was limited in the HCl-induced ALI (Fig. 1C). Inhibition of induction of edema by TP antagonism may be insufficient to restore the impaired gas exchange induced by the direct epithelial damage.

The LPS instillation did not induce lung edema. Supporting our hypothesis that TXA_2_ production may be responsible for edema in ALI, PGE_2_ but not TXB_2_ was detected in these lungs. In contrast to our observations, previous reports showed that LPS challenge induces edema in mouse lung^27^,^28^,^29^. We also previously showed that the LPS instillation caused lung hyper-permeability^16^. This discrepancy may be because of the differences in the time points selected for observation. In the present study, we examined symptoms at 6 h after the LPS treatment while the other studies made the observations 24 h or later after the treatment. We selected the 6 h-LPS instillation which is enough to cause neutrophil accumulation but not for induction of edema and lung dysfunction. There is a possibility that the longer period of stimulation with LPS may cause lung edema accompanied by TXA_2_ production.

The current study demonstrates that TP antagonism abolished the hyper-permeability and induction of edema, but it did not affect neutrophil migration in the lungs. We also confirmed that a strong migratory factor leukotriene B_4_, but not TXA_2,_ stimulates neutrophil migration activity *in vitro* (Fig. S8). These observations support the idea that TXA_2_ exacerbates ALI by disrupting endothelial barrier without influencing neutrophil activity. However, the vascular hyper-permeability is an initial step in neutrophil infiltration into the inflamed tissue. Infiltrated neutrophils produce proteases and reactive oxygen species, which in turn destroy endothelial barrier and induce further edema. Thus, it is difficult to clearly dissociate these two phenomena *in vivo*.

Although we here focused on the role of TXA_2_, the HCl administration elevated the amount of PGE_2_ in the BAL fluid (Fig. 1E). Previous studies showed that PGE_2_ administration enhanced endothelial barrier resulting in relief of symptoms in the LPS-induced ALI^30^. PGE_2_ produced may counteract excessive inflammatory response by reducing endothelial permeability and inflammatory cell infiltration in ALI.

Endothelial barrier disruption increases the leakiness of the vascular wall. In addition, we and others showed that tissue blood flow is the important factor of vascular permeability. The increase in tissue blood flow resulting from vasodilation elevates intravascular hydrostatic pressure, which intensifies outward transfer of serum components. Several physiological substances differently and uniquely modulate these two factors and regulate vascular permeability *in vivo*. Histamine induces vascular hyper-permeability through both blood flow increase and endothelial barrier disruption^31^. PGE_2_ increases tissue blood flow and induces vascular hyper-permeability while it enhances endothelial barrier integrity^32^. We have shown herein that TP-stimulation caused hyper-permeability in the skin ear capillary vessel. TXA_2_ analog U46619 reduced ear blood flow by inducing vasocontraction, while it disrupted endothelial barrier of microvessels (Fig. 4G,I). Lungs are composed mainly of endothelial cell-rich capillary vessels, which seem to be more sensitive to TXA_2_. Excessive TXA_2_ production can cause significant hyper-permeability resulting in severe edema in the ALI.

TXA_2_ is known to increase endothelial monolayer permeability in bovine aortic endothelial cells and HUVECs^33^,^34^. Consistently, we showed that endothelial TP agonism disrupted endothelial barrier in HUVECs. TP receptor is G_q_- and G_12/13_-protein coupled receptor and its activation elevates intracellular Ca^2+^ concentration and Rho activity^18^. Ca^2+^ increase and Rho activation induces actin cytoskeletal rearrangement such as stress fiber formation and myosin phosphorylation resulting in endothelial barrier disruption^35^. As expected, blockade of these signaling pathways impaired TP-mediated stress fiber formation and endothelial hyper-permeability (Fig. 5). These results allowed us to conclude that TP agonism increased endothelial permeability through Ca^2+^/Rho-mediated actin rearrangement.

In summary, the present study shows that TXA_2_ exacerbates ALI mainly by causing lung edema and that TXA_2_-TP signaling induces endothelial hyper-permeability through Ca^2+^/Rho signaling. These findings might help in understanding the pathological process of ALI, and provide new insight for therapeutic application of TP antagonists. We have utilized three different murine ALI models; HCl-, LPS + OA-, and LPS-induced ALI, which mimic acid aspiration-, lipid embolism-, or sepsis-induced human ALI. There are several other causative factors of ALI including mechanical ventilation and blood transfusion. Further detailed examination of each model and each disease stage will be indispensable to develop better therapy against ALI/ARDS.

## Materials and Methods

### ALI Induction

All the experiments were approved by the Institutional Animal Care Use Committee of the University of Tokyo. All experimental methods were performed in accordance with the approved guidelines. Eight-week old male C57BL/6J mice or BALB/c mice were purchased from CLEA Japan, Inc. (Tokyo, Japan). Mice were anesthetized with 3% isoflurane and maintained with 2% isoflurane. To make the HCl- or LPS-induced ALI model, HCl (0.1 M, 2.5 μl/g) or LPS (3.75 mg/kg) was intra-nasally administrated to C57BL/6J mice. To make LPS + OA-induced ALI model, LPS (1.5 mg/kg) was intra-nasally administrated, and 3 min later, OA (0.15 ml/kg) was intravenously injected into C57BL/6J mice. Six hours after the HCl or LPS instillation, mice were anesthetized and utilized for saturation of peripheral oxygen (SpO_2_) measurement or computed tomography analysis. For some experiments, the mice were euthanized and lungs were immediately perfused with normal physiological saline solution (33 mmHg).

### Computed Tomography Analysis

Mice were put into the chamber of the micro-CT system (LaTheta LCT-200, Aloka, Tokyo, Japan). X-ray voltage was 50 kV, the field of view was 48 × 48 mm, and the interval of scanning was 192 μm. The data were analyzed by the software provided by LaTheta.

### Saturation of peripheral oxygen (SpO_2_) Measurement

A mouse pulse oximeter (STARR Life Sciences, Oakmont, PA, USA) was used to monitor SpO_2_. The MouseOX collar clip was put on the neck of the anesthetized mice. After 3–4 min, the readings of pulse oximetry were recorded. The rate of sampling was once a second.

### Measurement of PGE_2_, PGI_2_ and TXB_2_

Mice were euthanized and 1 ml of total bronchoalveolar lavage (BAL) fluid was collected. After centrifugation (4 °C, 5 min, 500 × g), supernatant was collected. As internal standards, prostaglandin E_2_-d4 (PGE_2_-d4), 6-keto prostaglandin F_1α_-d4 (6-keto-PGF_1α_-d4) and thromboxane B_2_-d4 (TXB_2_-d4) were added to each sample. The samples were purified by Sep-Pak C18 3 cc Vac Cartridge (Water Corp., Milford, MA, USA). The quantification was performed using an LC/MS-8030 Triple Quadrupole Mass Spectrometer (Shimadzu, Kyoto, Japan) equipped with an electrospray interface.

### Morphological Analysis

The lungs from the experimental mice were fixed in 4% paraformaldehyde (PFA) for 24 h. Four μm-thick paraffin sections were stained with hematoxylin and eosin (H&E). For immunostaining, frozen 4 μm-thick sections were incubated with 0.3% Triton X-100 and 3% normal goat serum for 30 min, the sections were incubated with rat anti-mouse CD324 (1:200) and rabbit anti-thromboxane synthase antibody (1:100) overnight at 4 °C. After labeling with secondary antibodies, the samples were observed under a fluorescence microscope (Eclipse E800, Nikon, Tokyo, Japan). Some sections were used for chromogenic reaction with 3, 3′-diaminobenzidine (DAB) and observed under a light microscope (Optiphot-2, Nikon, Tokyo, Japan).

For whole-mount immunostaining of ear, mice were euthanized and perfused with 4% PFA. The ears were excised and ventral part of the skin was removed. The samples were permeabilized with 0.3% Triton X-100 for 30 min and blocked with 3% bovine serum albumin for 30 min. For cell staining, human umbilical vein endothelial cells (HUVECs, Lonza, Basel, Switzerland, passage 4–8) were seeded on glass coverslips. After the fixation with 4% PFA for 5 min, the cells were incubated with 0.1% Triton X-100 and 3% bovine serum albumin for 30 min. The ear or cell samples were incubated with goat anti-VE-cadherin antibody (1:200) overnight at 4 °C, and then incubated with rhodamine-phalloidin (3:100) at room temperature for 1 h. After labeling with secondary antibodies, the samples were observed under the fluorescence microscope.

### Water Content Measurement

Excised lungs were weighed to obtain the wet weight. The lungs were dried at 50 °C in an oven for 48 h, and weighed to get the dry weight. The lung water content was calculated by dividing the wet lung weight by the dry lung weight.

### MPO Activity Measurement

Excised lungs were homogenized in potassium phosphate buffer: 5 mM K_2_HPO_4_, 45 mM KH_2_PO_4_ (pH 6.0) with 0.3% hexadecyltrimenthyl ammonium bromide. After centrifugation (4 °C, 30 min, 20,000 × g), the supernatant was collected. The supernatant was incubated with 0.5 mM o-dianisidine dihydrochloride and 0.05% hydrogen peroxide for 5 min. MPO activity was measured as absorbance at 460 nm using a spectrophotometer (ARVO-SX 1420, PerkinElmer Japan, Kanagawa, Japan).

### Modified Miles Assay

U46619 (20 or 200 μg diluted in methyl acetate) was applied onto the right auricle of BALB/c mice. Evans Blue (50 mg/kg) was immediately administered intravenously. After 30 min, mice were euthanized and ears were excised, dried and weighed. The samples were then incubated with formamide in the 50 °C oven. Evans Blue extracted in formamide was quantified as absorbance at 610 nm, using the spectrophotometer.

### Intravital Microscopy

Fluorescein isothiocyanate–dextran (FITC-dextran, 66,100 Da) was injected (40 mg/kg) intravenously into BALB/c mice. U46619 (20 μg diluted in methyl acetate) was applied onto the auricle. The vasculature of mice ear was observed every 5 min with confocal microscope (ECLIPSE Ti with C1 confocal system, Nikon, Tokyo, Japan). FITC-dextran leakage and vascular diameter of arteries and veins were quantified as described previously with EZ-C1 FreeViewer (Nikon, Tokyo, Japan)^32^.

### Blood flow measurement

U46619 was applied onto the auricle of BALB/c mice. Changes in ear blood flow were monitored for 1 h by using an Omegazone laser Doppler blood-flow imaging system (Omegawave, Inc., Tokyo, Japan). Blood flow was quantified for each experimental mouse after 20 min of stimulation, and expressed as the difference in intensity between the right and left ear.

### Transwell Permeability Assay

HUVECs (8 × 10^4^) were seeded onto the upper inserts of transwell (1 μm pore size, BD Biosciences, Bedford, MA, USA) and grown to confluent. After 4 h starvation with 2% fetal bovine serum, U46619 (0.1–10 μM) was administrated into the upper inserts, and then 70,000 Da FITC-dextran (24 μg per 300 μl of medium) was added to the upper inserts. FITC-dextran passed through the cell monolayer was collected every 10 min. The amount of FITC-dextran was measured by the fluorescence spectrophotometer using excitation/emission wavelengths of 485/535 nm.

### Transendothelial Electrical Resistance (TER) Measurement

TER was measured using an xCELLigence Real-time Cell Analyzer DP system (Roche, Indianapolis, IN, USA). HUVECs were seeded onto electrodes and grown until confluent. After the serum starvation, U46619 (0.1–10 μM) was administrated and TER was measured every 1 min. For the normalization, TER values are expressed as the ratio to the initial value at 30 min before the U46619 administration.

### Reagents

The reagents that we used are given below: SQ29548 and U46619 (Cayman chemical, Ann Arbor, MI, USA); rat anti-mouse CD324 (Biolegend, San Diego, CA, USA); rat anti mouse CD41 (AbD Serotec, Oxford, UK); rabbit anti-thromboxane synthase antibody (Abcam, Cambridge, UK); goat anti-VE-cadherin antibody (Santa Cruz, Dallas, Texas, USA); rhodamine-phalloidin (Life Technologies, Carlsbad, CA, USA); Escherichia coli lipopolysaccharide O55:B5, 66,100 Da FITC-dextran, and 70,000 Da FITC-dextran (Sigma-Aldrich, St. Louis, MO, USA); Ozagrel (LKT laboratories, Inc., St. Paul, MN, USA); Y27632 (Merck Biosciences, Darmstadt, Germany); LaCl_3_ (Nakarai tesque, Kyoto, Japan); and histamine dihydrochloride (Wako, Osaka, Japan).

### Statistical Analysis

The results of the experiments were expressed as means ± S.E.M. Statistical evaluation of the data was performed by student’s t test for comparison between two groups and by one-way ANOVA followed by the Turkey test for comparison between more than two groups. P < 0.05 was taken as significant.

## Additional Information

**How to cite this article**: Kobayashi, K. *et al.* Thromboxane A_2_ exacerbates acute lung injury via promoting edema formation. *Sci. Rep.* **6**, 32109; doi: 10.1038/srep32109 (2016).

## Supplementary Material

Supplementary Information
