# Supplementary material for: Thromboxane A2 exacerbates acute lung injury via promoting edema formation
Source: Sci Rep. 2016 Aug 26;6:32109. doi: 10.1038/srep32109 (PMC4999811; doi:10.1038/srep32109)
Supplement: Supplementary Information [file srep32109-s1.pdf]

Thromboxane A_2 exacerbates acute lung injury via promoting edema formation

Koji Kobayashi, Daiki Horikami, Keisuke Omori, Tatsuro Nakamura, Arisa Yamazaki, Shingo Maeda, Takahisa Murata.

Online Data Supplement

Supplemental figures

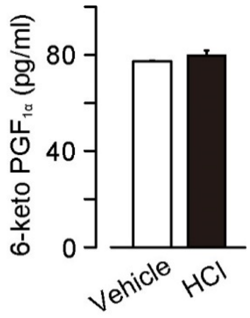

Supplemental Figure S1. HCl-administration did not induce PGI_2 production. 6-keto $PGF_{1\alpha}$ content in BAL fluid of vehicle- or HCl (intra-nasally, 0.1 M, 2.5 μl/g, 6 h)-administrated mice (n=5-6). Data are presented as mean ± SEM.

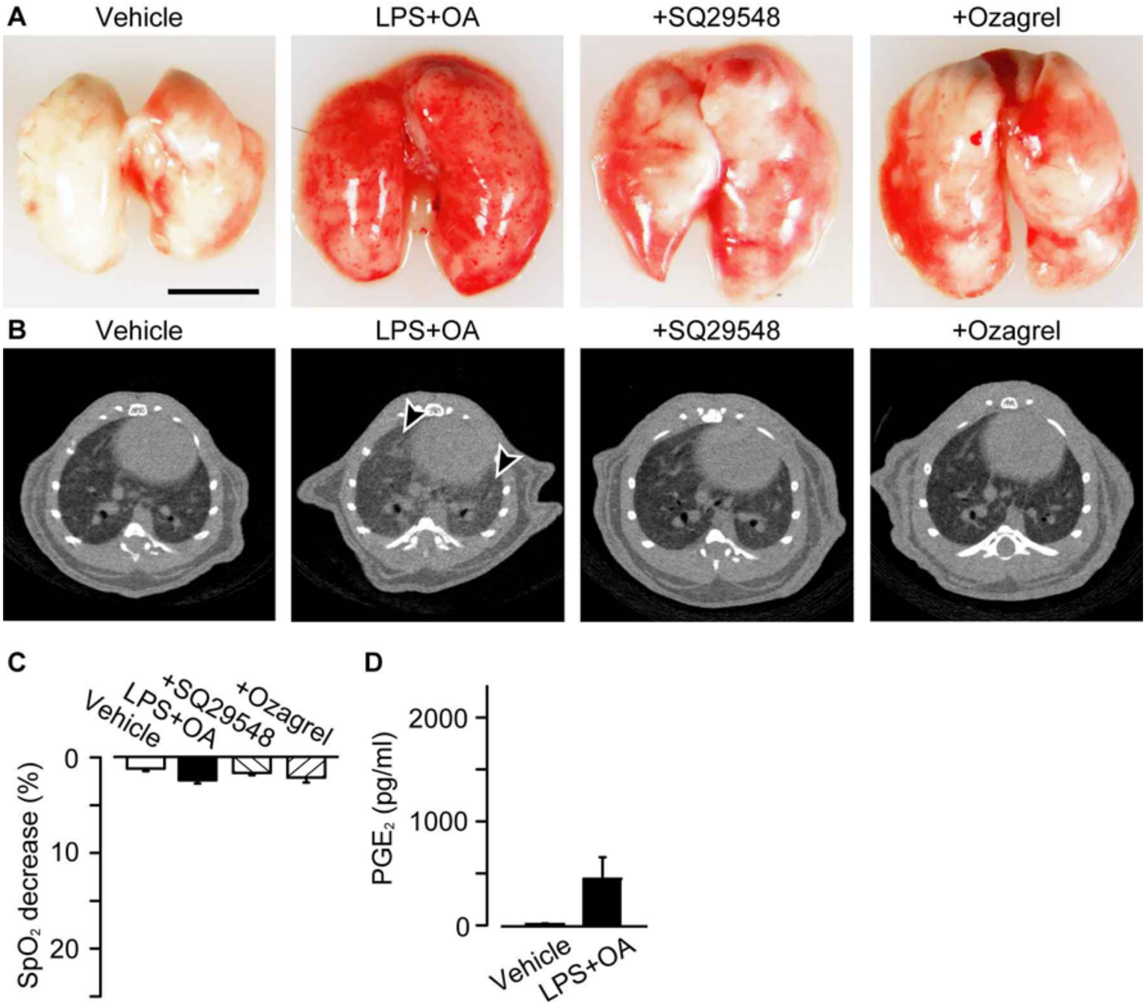

Supplemental Figure S2. TXA_2-TP signaling abrogates LPS+OA-induced ALI model. SQ29548 (i.p., 2 mg/kg, 0, 2, 4 h after LPS administration) or Ozagrel (i.p., 50 mg/kg, 15 min before OA administration) was administered to the OA (i.p., 150 µl/kg) and LPS (intra-nasally, 1.5 mg/kg, 6 h)-treated mice. (A) Representative pictures of lungs. Bar, 5.0 mm. (B) Representative pictures of CT scan, arrow head indicates infiltrative shadow). (C) Values of saturation of peripheral oxygen (SpO_2) in the mice (n=4-6). (D) PGE_2 content in lung BAL fluid (n=5). Data are presented as mean ± SEM.

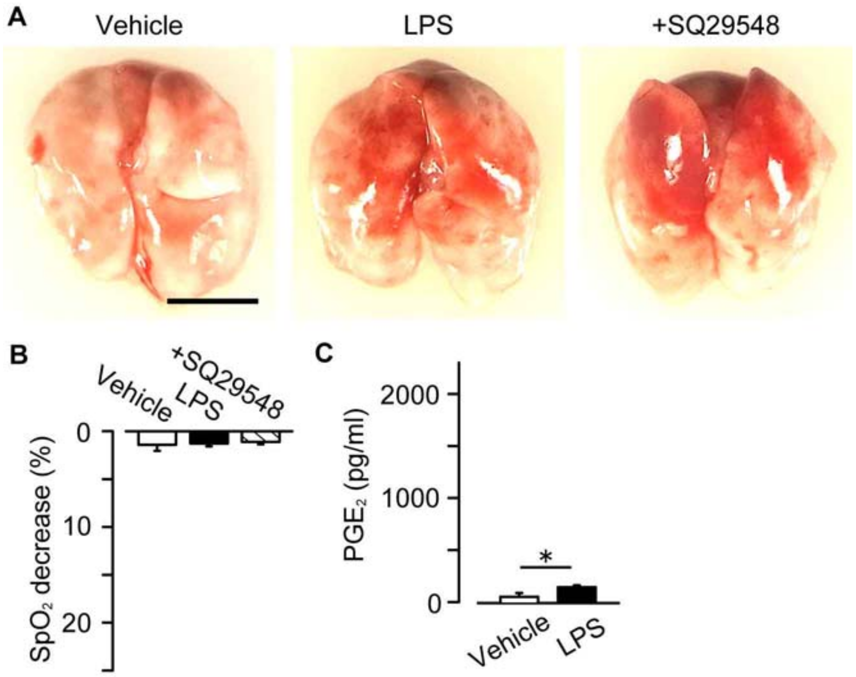

Supplemental Figure. S3 TXA_2-TP axis did not influence the LPS-induced ALI. SQ29548 (i.p., 2 mg/kg, 0, 2, 4 h after LPS administration) was administered to LPS (intra-nasally., 1.5 mg/kg, 6 h)-treated mice. (A) Representative pictures of lung. (B) Values of saturation of peripheral oxygen (SpO_2) in the mice (n=4-6). (C) PGE_2 content in lung BAL fluids (n=5). Data are presented as mean ± SEM. *P<0.05.

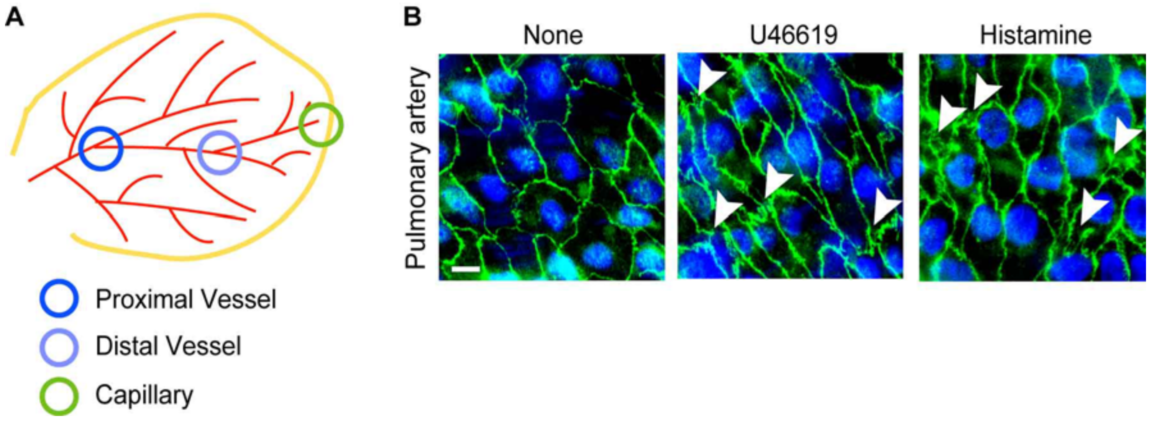

Supplemental Figure S4. TP stimulation causes endothelial barrier disruption *ex vivo*. (A) Illustration of vascular structure in mouse ear. (B) En face immunostaining of mouse pulmonary artery was performed as previously described.[1] Representative pictures of en face immunostaining of VE-cadherin in mouse pulmonary artery. Bar, 10 μm.

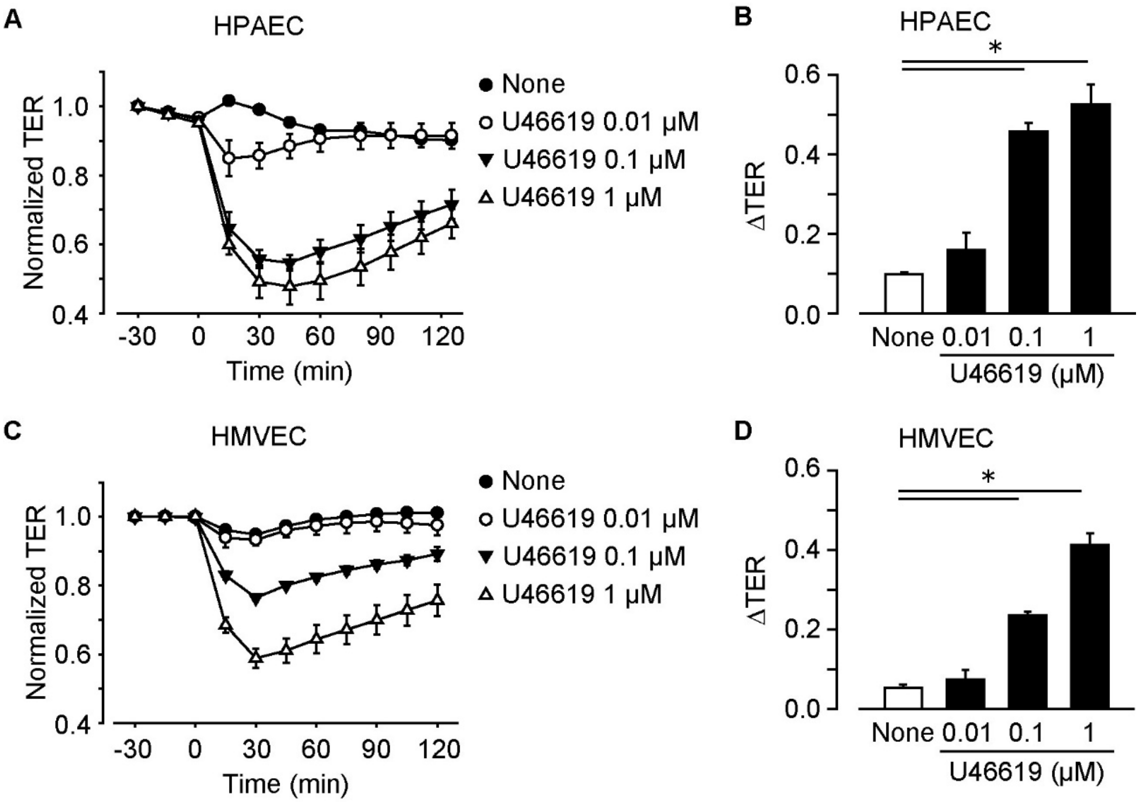

Supplemental Figure S5. TP stimulation induced endothelial barrier disruption in HPAECs and HMVECs. TP agonist (U46619, 0.01-1 μM) was administrated onto cells and TER was measured every 15 min. (A) TER of human pulmonary endothelial cells (HPAECs). (B) Summary of the maximum decrease of TER (n=4). (C) TER of human microvascular endothelial cells (HMVECs). (D) Summary of the maximum decrease of TER (n=4-6). Data are presented as mean ± SEM. *P<0.05.

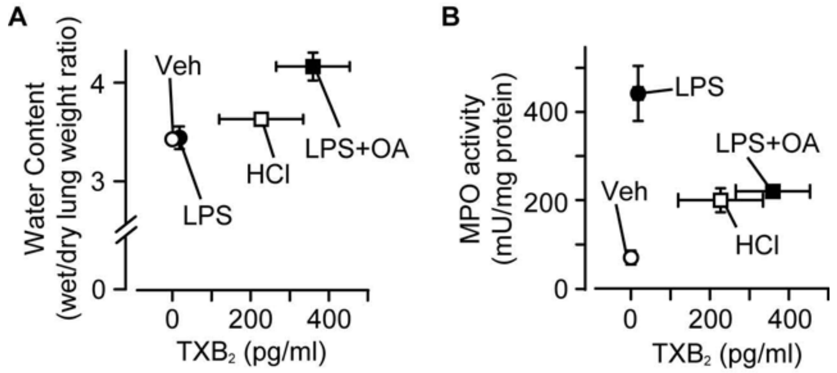

Supplemental Figure S6. The level of edema formation is positively correlated with TXA_2 production.

(A) Scatter plots of water content vs TXB_2 production in BAL fluids (n=4-15). (B) Scatter plots of myeloperoxidase (MPO) activity vs TXB_2 production in BAL fluids (n=4-15). Data are presented as mean ± SEM.

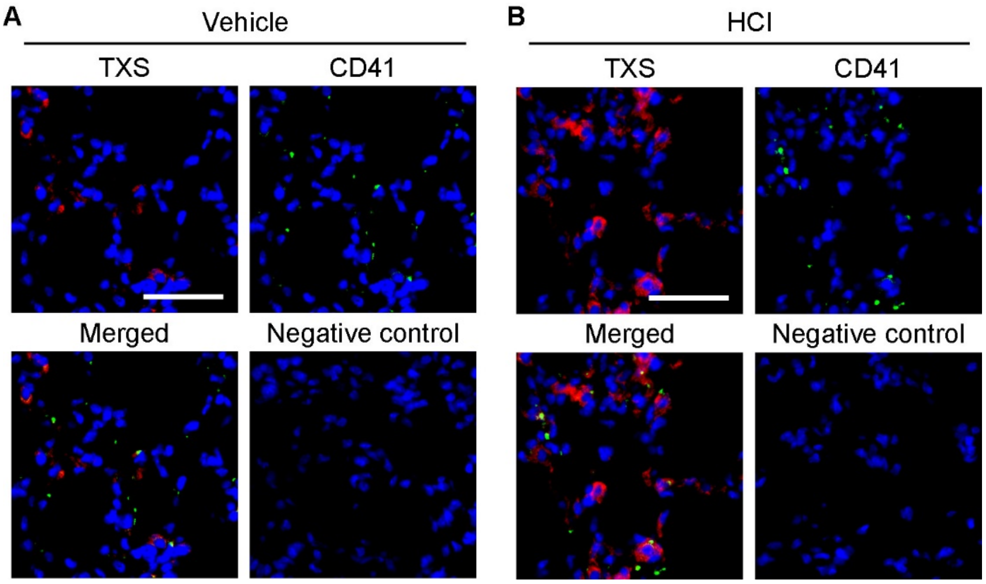

Supplemental Figure S7. TXS was not expressed on CD41 positive cells in lung. Representative pictures of immunostaining of TXS (upper left panel, red) and CD41 (upper right panel, green) of vehicle (A) or HCl (B; intra-nasally, 0.1 M, 2.5 μl/g, 6 h)-administrated mouse lung. Lower left panel shows merged pictures of TXS and CD41 staining. Lower right panel shows negative controls which only treated with secondary antibodies. Bar, 40 μm.

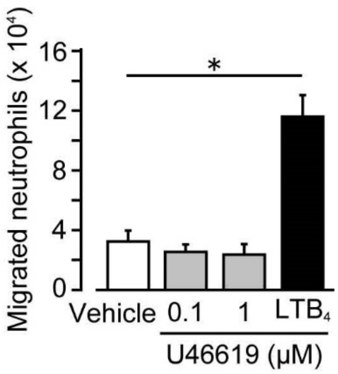

Supplemental Figure S8. TP stimulation did not induce neutrophil chemotaxis. Neutrophil chemotaxis assay was performed as previously described.[2] A modified Boyden chamber with 8-µm pore (BD Biosciences, Bedford, MA, USA) was used. Stimulants were added to the bottom chamber. 2×10^5 isolated neutrophils were applied to the upper inserts. After 1 h incubation with RPMI 20 mM HEPES 10% FBS, the number of cells were counted (n=6). Data are presented as mean ± SEM. *P<0.05

References

1 Omori, K., Kida, T., Hori, M., Ozaki, H. & Murata, T. Multiple roles of the PGE_2 -EP receptor signal in vascular permeability. *British journal of pharmacology* **171**, 4879-4889, (2014).

2 Murata, T. *et al.* Anti-inflammatory role of PGD_2 in acute lung inflammation and therapeutic application of its signal enhancement. *Proceedings of the National Academy of Sciences* **110**, 5205-5210, (2013).
